# Diverse approaches to nature recovery are needed to meet the varied needs of people and nature

**DOI:** 10.1007/s11625-023-01337-w

**Published:** 2023-05-25

**Authors:** Rowan Dunn-Capper, Laura C. Quintero-Uribe, Henrique M. Pereira, Christopher J. Sandom

**Affiliations:** 1 German Centre for Integrative Biodiversity Research (iDiv) Halle-Jena-Leipzig, Puschstrasse 4, 04103 Leipzig, Germany; 2 Institut für Biologie, Martin-Luther-University Halle-Wittenberg, Halle, Germany; 3 CIBIO (Research Centre in Biodiversity and Genetic Resources)–InBIO (Research Network in Biodiversity and Evolutionary Biology), Universidade do Porto, Vairão, Portugal; 4 School of Life Sciences, University of Sussex, Brighton, BN1 9QG UK; 5 Sussex Sustainability Research Programme, University of Sussex, Brighton, BN1 9QG UK

**Keywords:** Nature futures framework, Multicriteria mapping, Rewilding, Ecological restoration, Stakeholder values

## Abstract

Conservation and restoration projects often fail to engage local communities during the planning and implementation stage. In addition, when considering urban boundary ecosystems, there exists a wide range of stakeholders that must be involved in the planning process to ensure social equity in land management outcomes. Traditional methods for assessing future landscape change scenarios have been critiqued for their inability to adequately incorporate the diverse range of stakeholder values. This paper presents a multicriteria mapping study, incorporating a novel application of the Nature Futures Framework, to assess nature recovery scenarios on Brighton and Hove’s Downland Estate—an urban boundary landscape surrounding the city of Brighton and Hove in Sussex, South East England. We focus on two key research outcomes. First, we assess the perceived performance of alternative nature recovery options across Nature Future value perspectives and between contrasting stakeholder groups. Second, by mapping stakeholder values from our multicriteria mapping study, we demonstrate that the Nature Futures Framework provides a robust framework within which to assess the diverse values stakeholders hold for land use change. We propose that utilizing the Nature Futures Framework, in combination with the multicriteria mapping interview technique, can form a valuable tool to elicit stakeholder values that may have been hidden, or underrepresented in traditional assessment methods, and to compare the perceived performance of alternative nature recovery scenarios between stakeholder groups.

**Supplementary Information:**

The online version contains supplementary material available at 10.1007/s11625-023-01337-w.

## Introduction

In Europe, urban and peri-urban systems form important sites for agricultural production and provide goods and services from the local to the global market (Opitz et al. 2016). Given that we have entered the UN Decade on Restoration (UNEP/FAO 2019), there exists the opportunity to focus land management towards nature recovery, for example through targeted restoration or rewilding, to create alternative societal and environmental benefits. While agricultural intensification is one of the main drivers of biodiversity loss worldwide (Norris 2008; Henle et al. 2008), managed agricultural landscapes are also culturally important (Van Berkel and Verburg 2014). When considering changes in land management, we need to account for the diverse range of stakeholder values at a given site. This is particularly important in the peri-urban context where numerous stakeholders are likely to be directly affected by changes in the landscape.

It has been widely acknowledged in the literature that conservation and restoration projects often fail to engage local communities during the planning stage (Pereira et al. 2020; Pascual et al. 2021; Fischer et al. 2021), which can give rise to novel conflicts that could alter the status quo between human–nature relationships (Killion et al. 2021). There is a growing need to develop policy approaches that better address the disconnect in how nature is valued by different stakeholder groups (Pascual et al. 2021). It is thus important for land management planning to promote inclusive participation that better accounts for the diverse values people attach to nature (Anguelovski et al. 2020; Langemeyer and Connolly 2020). Here, participatory techniques can better incorporate people into decision-making processes.

Multicriteria mapping (MCM) is a participatory interview technique which can be used in landscape planning to reveal stakeholder priority values and outcomes for land management as well as exploring their perspectives on how different land management options will perform in delivering them. Involving multiple stakeholders in landscape planning processes helps to identify potential areas of conflicts or co-benefits between nature and people, and studies have described successful initiatives where the involvement of local stakeholders resulted in innovative solutions for nature recovery (e.g. Heikkinen et al. 2012; den Herder et al. 2017). The MCM approach explicitly opens up understanding of stakeholders' values and allows for the comparison of how different land use change scenarios might deliver against stakeholders’ diverse values and needs.

Ecosystem service-based assessments have been widely applied to assess the benefits ecosystems provide society (Millenium Ecosystem Assessment 2005) and have become increasingly popular within a prominent section of the conservation movement (Pascual et al. 2021). While the ecosystem service approach has diversified to include broader societal and cultural values of nature, it still has a focus on use values—the direct and indirect ways nature benefits people (Kenter et al. 2015, 2019; Chan et al. 2018). Chan et al. (2016, 2018) highlight the need to engage with a wider range of values to better represent the ways in which people relate to nature. The Nature Futures Framework (NFF) was developed under the Intergovernmental Science-Policy Platform on Biodiversity and Ecosystem Services (IPBES) as a scenario tool to better capture the diverse values humans hold for nature (Pereira et al. 2020).

**Fig. 1:**
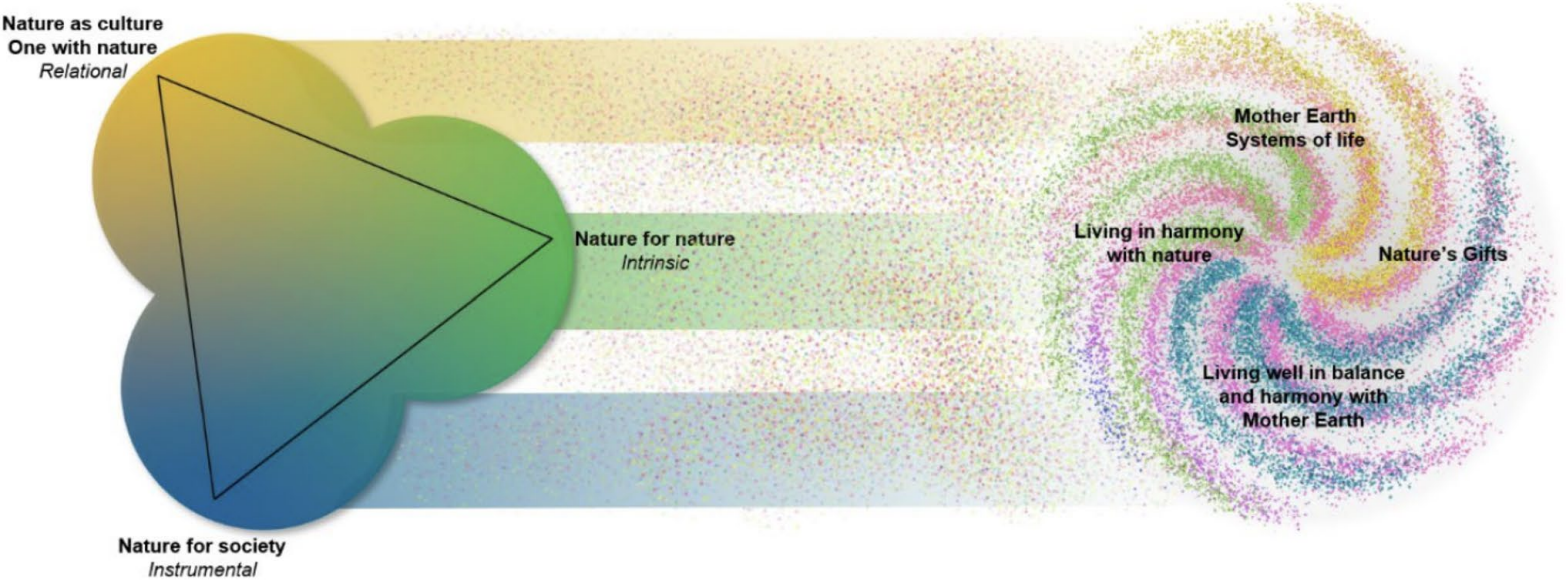
The Nature Futures Triangle (left) (from IPBES 2022), presents the three value perspectives of nature (NC, NN, NS)

The NFF is a “flexible tool to support the development of scenarios and models of desirable futures for people, nature and Mother Earth” (IPBES 2022) that aims to allow the exploration of alternative pathways in which people and nature can interact. Developed to help meet the UN Sustainable Development Goals (Rosa et al. 2017) and the post-2020 global biodiversity framework (CBD 2022), the key foundations of the NFF are to incorporate the multiple value perspectives of nature and the critical feedbacks of socio-ecological systems (Kim et al. 2021). In contrast to more traditional approaches, such as ecosystem services, the NFF explores use values, as well as non-use values (including intrinsic values) and relational values (Kim et al. 2021; Mansur et al. 2022). This makes it particularly useful when assessing different visions on nature conservation and restoration (Quintero‐Uribe et al. 2022). The approach can be visualized using the Nature Futures Triangle (Fig. 1).

The NFF can be combined with participatory scenario approaches to include social practices into value articulation, allowing us to better incorporate the multidimensionality of stakeholder values for nature into decision-making (Ernstson 2013; Kenter et al. 2015; IPBES 2022). In this study, we present an alternative novel application of the NFF as a tool to assess alternative future nature recovery options for the Downland Estate. Instead of using the NFF to build and classify scenarios, we use it map the diverse values stakeholders hold for alternative nature recovery options. By combining MCM with the NFF we explore a new approach to landscape planning, that incorporates the diverse values of stakeholders.

In Brighton and Hove, South East England, the land management of the city’s surrounding Downland Estate is currently under review (Brighton and Hove City Council 2021). While current use is largely tenant farming, there exists potential to alter management towards alternative nature recovery trajectories. The Downland Estate is situated within “The Living Coast”, a 390 km^2^ UNESCO Biosphere Reserve within which over 300,000 people live (The Living Coast 2021). Biosphere reserves aim to foster both social and economic development alongside biodiversity conservation (Ferreira et al. 2018), and provide test sites for managing changes and interactions between social and ecological systems (UNESCO 2021). Thus, the Downland Estate provides the ideal context for studying the social values of landscape change. This study uses the participatory multicriteria mapping (MCM) interview technique, in combination with the NFF, to highlight stakeholder values for alternative future nature recovery options, with the overarching aim of attempting to better understand how people value changes.

The aims of this study were twofold: first, we use the MCM methodology to assess different nature recovery options for the Downland Estate, assessing the underlying motivators of participant preferences across and between stakeholder groups. Second, we explore the capacity of the NFF to incorporate pluralistic values (IPBES 2022) by using the framework to map stakeholder values of landscape change.

## Methods

### Case study area

The Brighton and Hove Downland Estate is a 5,200-ha area surrounding the city of Brighton and Hove in East Sussex, England. The entire site is characterized by chalky, silty loam soil type of intermediate depth (Brighton and Hove City Council 2022). Current land use across the site is 78% farmland, with 9% woodland. There are 40 wildlife sites, 5 local nature reserves, 2 sites of special scientific interest, 1 special area of conservation, and 1 national nature reserve. Primarily, the land consists of grade 3 (good to moderate) and grade 4 (poor quality) agricultural land. At the time of research, the city council were actively developing a new whole estate management plan (Brighton and Hove City Council 2021) to support future decision-making across the breadth of the site; thus the study was especially pertinent to stakeholders, as the context of landscape change at the site was realistic.

### Multicriteria mapping

**Fig. 2:**
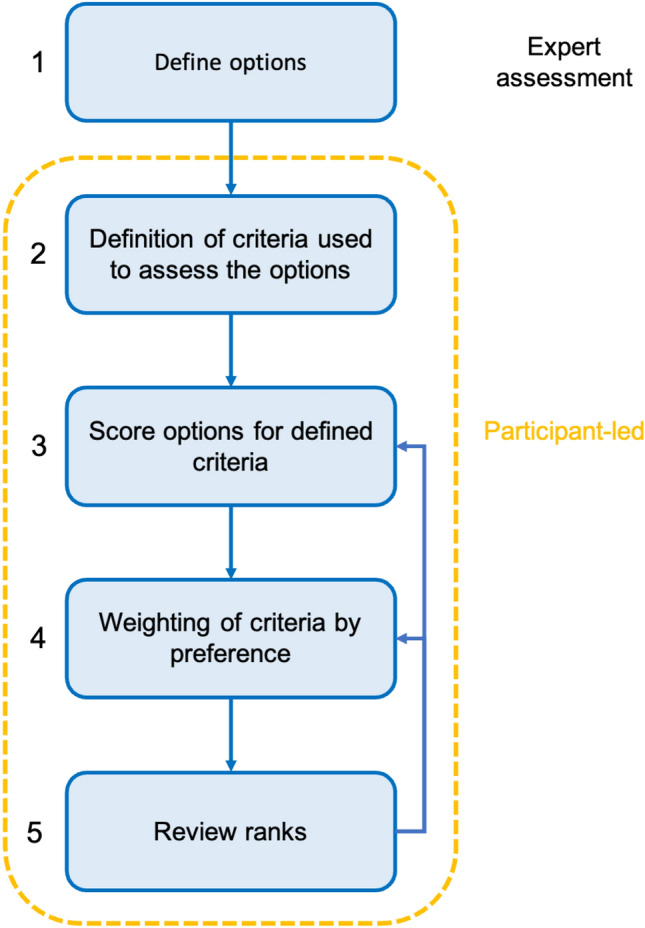
The five key stages (labelled) of the MCM methodology, for participant-led assessment of different landscape options

Multicriteria Mapping is a participatory social appraisal tool (Durrant and Ely 2022) that aims to “put the participant in the driving seat” (Coburn et al. 2019). It can be used to elicit participant values which may be overlooked in conventional approaches, and to create an overall ranking of the assessed scenarios against their perceived ability to deliver participant values. While MCM is structured, following pre-defined stages, emphasis is also placed on flexibility and creativity (Coburn et al. 2019). The methodology relies on a dedicated web-based software tool^1^ to collect and analyse data. MCM relies on five key stages (Coburn et al. 2019) (select options > define criteria > assess scores > assign weights > review ranks) (Fig. 2), outlined below.

In the first stage of an MCM appraisal, the researcher defines *options* (Fig. 2, Stage 1) they would like the participant to evaluate. Options must be clearly defined, and are typically provided to the participants in advance of the interview. All participants will evaluate all options that have been selected by the interviewer, it is thus important they offer a comprehensive range of scenarios in the given context. If they believe any options have been missed, participants are also free to define their own options. Additional options are only included in the appraisal of the participant that defined them.

**Fig. 3:**
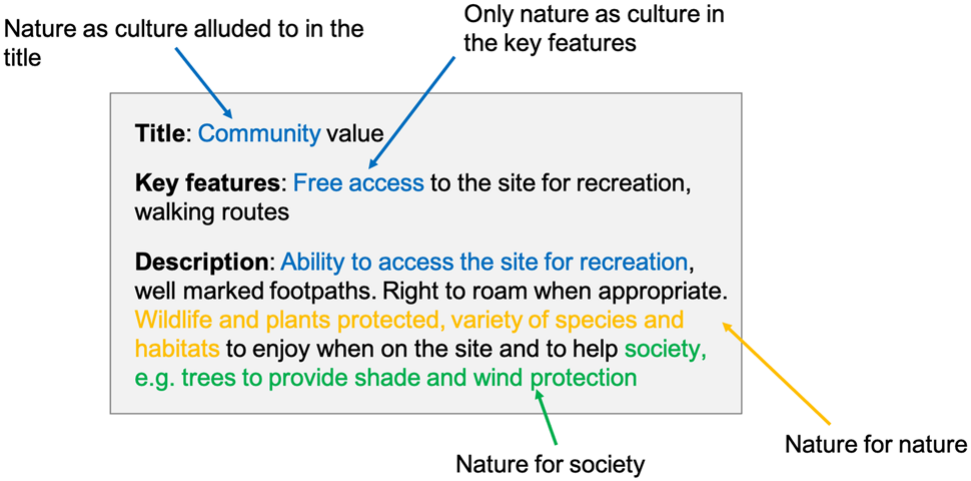
Scoring process for a hypothetical criterion “Community Value”. There are only NC elements in the title and key features, and this is therefore the main NFF perspective. However, there are elements of NN and NS in the description. A possible scoring for this criterion would be 0.8 NC, 0.1 NN, 0.1 NS. This should be discussed by the individual scorers before assigning a final score

Participants are then asked to define *criteria* with which to assess the options (Stage 2). Criteria are personal to each interviewee, and are the key factors they have chosen to assess the pros and cons of the different options (Coburn et al. 2019). Participants are free to define as many criteria as they see appropriate; however, it is suggested they initially define 3–5 criteria. More criteria may then be added depending on time constraints. For each of their individual criterions, the participant must provide a title, key features and description, which are inputted into the MCM software (an example criterion with title, key features, and description is presented in Fig. 3).

Following definition of their criteria, the participant is then asked to score the perceived performance of their criteria against each option (Stage 3). While participants are free to use any scale they see fit (which is accounted for during the software analysis), a range of 0–100 is typically used—where 0 is worst-possible performance and 100 is best possible performance. To account for uncertainty in option performance, participants assign a pessimistic (the perceived worst-case performance of the land management option under this criterion), and an optimistic score (the perceived best-case performance of the land management option under this criterion). For example, a participant assessing the regenerative agriculture option for the hypothetical criterion “Community value” may assign a low pessimistic score (e.g. 10) on the basis that the public could be excluded from the site, but a high pessimistic score (e.g. 90), since local people could be actively involved.

Finally, after scoring all the options for all of their criteria, the participant assigns weights to their criteria (Stage 4). This stage is important to reflect the fact that participants may value their criteria differently when making overall decisions. The criteria they perceive to be more important are assigned higher weights. For example, a participant may have defined three criteria; however, they perceive the performance of the options under one specific criterion to be significantly more important than for the other two criteria when making an overall assessment of the best option. Therefore, this stage of the process allows the participant to reflect this preference in the scoring by weighting this criterion higher than the other two criteria. The software uses the criteria scores and weightings to generate a figure showing the participants’ overall rankings for each option. The participant is free to review these ranks (Stage 5) and may reassess scores and weightings if they feel strongly about specific outcomes.

### Downland Estate MCM

We conducted our MCM with the aim of eliciting stakeholder values for nature recovery on the Downland Estate. A local expert, actively involved in the consultation process, helped identify suitable participants for this study. Participants were selected to encompass two key stakeholder groups, producers and conservationists. These two groups were selected to try and highlight the dichotomy in values that may be present when assessing future land uses in the peri-urban context, since we anticipated potential value conflicts between the groups. Participants from outside these groups were also approached if they were believed to possess an important and novel insight into nature recovery on the estate. Combined with internet searches to highlight potential interviewees, a total of 41 potential participants were approached. Contact with prospective participants was initially made by email in December 2021. If the respondent did not reply, a follow-up email was sent a fortnight later.

The study included 13 individuals, with five interviewees from each core stakeholder group. Three interviewees were assigned to the “other” group. These individuals were involved in education, research, and water management for the Estate and were selected to highlight alternative viewpoints and a broader range of values to the two core groups studied.

Participants assigned to the producer group were involved in either tenant farming on the Downland Estate, or community-based production on the Estate or within Brighton City. It was considered important to include participants outside the bounds of just traditional agricultural production, particularly focussing on those involved in community-based production, which is an important initiative within the Urban Nature Futures framework (Mansur et al. 2022). Individuals classified as conservationists were all involved in local organizations concerned with nature restoration in Sussex, and their interests ranged from rewilding to targeted restoration of chalk grassland.

Before the MCM interview, participants were issued with a detailed briefing document (see supplementary information, S1), outlining the MCM interview technique and the scenarios to be considered. In our study, participants were asked to assess landscape change for a hypothetical contiguous land holding on the Downland Estate with the following characteristics: 200-ha in size, currently being used for arable production, and within walking distance of the city outskirts. Next, participants were asked to assess four contrasting nature recovery *options* and one control *option* representing the status quo using their own personally defined *criteria*. In most cases, participants had read the briefing document in detail, and came prepared with criteria to use in the assessment.

The *options* used in this study (Table 1) were defined to be realistic land management alternatives for the Downland Estate, based on the previous work of Balfour et al. (2021) and additional expert assessments. The Traditional Family Farm option was a status quo option, as it aligned most closely with current land use on the Estate. There were two rewilding options: passive rewilding, a hands-off restoration technique that emphasizes natural ecosystem regeneration and processes (Pereira and Navarro 2015), which has been highlighted in the literature as a cost-effective mechanism for ecological restoration (Schou et al. 2021); and agricultural rewilding, a more specialized form of rewilding that fits closely with the nearby Knepp Estate, a 1,400-ha site, which is one of the most well-known rewilding projects in the UK (Dempsey 2021). The targeted restoration option was defined specifically for the Downland Estate, as chalk grassland is a highly valued and unique local habitat that community groups are actively advocating to be restored (e.g. Brighton Downs Alliance 2022). The regenerative agriculture option was defined as a more “environmentally friendly” production option, focussing on improving soil quality while also producing high-quality farm products (LaCanne and Lundgren 2018).

**Table 1:** Land management options

Nature recovery options	Description
Traditional Family Farm (status quo, this option best replicates current land use on the site)	This option is focussed on agricultural production. The site will be a mix of arable fields (primarily producing spring barley and winter wheat), with agrochemicals used to increase yield. Ideas of nature recovery are not at the forefront of land management plans All farming at the site complies with existing environmental standards (under Red Tractor certification)
Regenerative agriculture	Regenerative agriculture is an alternative means of food production. It is based on the following key processes: no, or low, external inputs, and increasing the efficiency of on farm inputs; integration of livestock in the agricultural system; no or minimal use of synthetic fertilizers and pesticides; and reduction in, or elimination, of tillage At this site, all livestock production will be certified to the Soil Association organic standard Alongside meat production, organic fruit and vegetable production, such as the planting of apple orchards, will be prioritized
Agricultural rewilding	This option comprises a significant shift towards a landscape governed by natural processes. Arable production at the site would cease, and free roaming heritage cattle and fallow deer are introduced, with the site boundary fenced (that allows public access through pre-established public rights of way). Seasonal introductions of heritage pigs Over time, human intervention would be minimal, with emphasis on allowing plant communities to develop naturally. Rather than working to prevent disturbance regimes, such as wind damage, these are accepted as natural processes that shape the landscape The opportunity for food production in this scenario is associated with the management of the introduced large mammal populations
Targeted restoration	This option is targeting the restoration of chalk grassland as a locally important habitat. Arable production at the site would cease. After the harvesting of the last crop a sterile seedbank is created by using a herbicide to remove arable weeds. The land is then cultivated and sowed with locally sourced native seeds Hay cropping in the second and third year of the restoration programme would be used to facilitate nutrient reduction in the soil and the flowering and seeding of chalk grassland flora. Managed grazing regimes would be implemented, primarily with sheep grazing in the autumn and winter. Cattle may also be used to achieve restoration goals if required
Passive rewilding	This option comprises a significant shift towards a landscape governed by natural processes, this means minimal human intervention and emphasis on allowing plant communities to develop naturally. There are no species reintroductions. Instead, a passive approach is taken to nature restoration Rather than working to prevent disturbance regimes, such as wind damage, these are accepted as natural processes that shape the landscape There is no agricultural production undertaken in this option; however, non-extractive businesses are possible, and recreation is welcomed

The quantitative scoring process was used to guide the participant through the MCM assessment and generate the ranking figures. The ranking data for the land management options was calculated within the MCM online software. A rank is the sum of the weighted scores given to the options by the participants. More information on the ranking procedure can be found in the MCM Manual (Coburn et al. 2019). MCM is not a conventional statistical analysis, and thus the smaller sample size was not seen as limiting. Instead, the aim is to try and understand the range of perceptions surrounding the future of the Downland Estate. Thus, the qualitative data elicited during the process was equally as important in the analysis of participant preferences.

Qualitative data were collected by the interviewer within the MCM software, which allowed annotations to be made next to each participant’s criteria definition and scoring. At the start of the interview, the alternative management options were discussed and the respondents’ general perception of each was noted. Next, the interviewer worked with the respondent to define their criteria, and any supplementary information outside of the criteria title, key features and description was noted. During the scoring and weighting stages, the respondents were encouraged to justify their scorings. Throughout the interview the participant was able to read the notes being made by the interviewer and were encouraged to ask the interviewer to modify or add to their notes if they believed any key information had been missed. Additionally, interviews were recorded and could be revisited to deal with any ambiguity in the notes.

Once all the scheduled interviews were concluded, the criteria were split between the NFF perspectives (NN, NS, NC) within the Multicriteria Mapping software (assigned to the highest scoring perspective from the NFF mapping). This allowed the performance of the options to be assessed for each individual NFF perspective. Ranks were calculated for each perspective using only the scores of criteria for a given NFF perspective. For this stage of the analysis, it is important to note that the absolute values calculated in the rankings reflect only the numerical values of the overall ranks for the included options so the absolute values of maxima will not be as high as the rankings that incorporate all criteria. What is important are the ordinal patterns, not the absolute values from the rankings.

The interviews were undertaken between January 2022 and March 2022 and were conducted online in accordance with COVID-19 guidelines at the time. All participants in the study gave written consent to participate, and approval was given by the University of Sussex Research Ethics Committee to carry out this research (ER/CS546/3).

### Nature Futures Framework mapping

Typically, the NFF is used in scenario development; however, our novel application allowed us to assess its flexibility as a framework in which to classify stakeholder values. The NFF descriptions used to categorize the criteria were taken from Kim et al. (2021), Mansur et al. (2022), Pereira et al. (2020), and Quintero‐Uribe et al. (2022):

1. Nature for nature (*intrinsic*)—criterion emphasizes preservation of biodiversity and nature for what it is.
2. Nature for society (*instrumental*)—criterion highlights the benefits people derive from nature, for example the provision of ecosystem services.
3. Nature as culture, one with nature (*relational*)—criterion relates to the reciprocal character of the human–nature relationship, which is the relationship that nature and people co-create. The focus is on concepts mentioning engagement with nature boosting social cohesion or cultural identity.

We first scored each of our 47 participant-defined criteria for the Nature Future value perspectives (NS, NN, NC) individually. The scoring was done by analysing the title, key features and description of each criterion that were defined by the participant and looking for elements in the text that aligned with the NFF perspectives. Each criterion was given a score for each NFF perspective between 0 and 1 (in increments of 0.1), where a value of 1 means the criteria fully matched with the given NFF perspective description. For each criterion, the combined score across perspectives always summed to one. For example, a criterion scoring 1 for NN would consequently score 0 for NS and NC, as it was perceived to solely include elements of the nature as society perspective, while a criterion scoring 0.6 for NN, 0.2 for NS, and 0.2 for NC is seen as mainly mapping to NN, but also containing some elements of NS and NC. Generally, reference to a specific NFF perspective in the criterion title was weighted highest in the scoring, reference in the key features was secondary in importance, and reference in the description was seen as least important.

The criteria were first scored individually (by RC and LQ) and then scorings were discussed collectively between researchers (RDC, LQ, CS). When there was a discrepancy in scoring or a criterion that had been identified as difficult to score or comprehend, this criterion was discussed in detail, and the key features and description worked through to finalize the score. In the rare case that more information on the participants’ values was needed, this was investigated by looking at the qualitative data—i.e. the participant’s rationale when assigning their scores during the MCM interview process. The NFF mapping for the 47 criteria from the Downland Estate interviews are summarized in Table 2. The scoring process for a hypothetical criterion “Community Value” is shown in Fig. 3.

**Table 2:** Every participant-defined criterion from the Downland Estate MCM

Criteria	Keywords	NN	NS	NC	Stakeholder group
Accessibility	Human enhancing Accessible to people Conscious and health benefits	0	0.3	**0.7**	Producer
Animal welfare	Welfare of animals in the area	**0.8**	0	0.2	Conservationist
Benefits to people	Water supplies Clean air Less chemical inputs Access to nature	0	**0.8**	0.2	Conservationist
Biodiversity	Diversity of flora and fauna Butterflies and pollinators	**0.9**	0.1	0	Producer
Biodiversity (2)	Species diversity	**1**	0	0	Other
Biodiversity education^a^	Make people aware of biodiversity loss Engage with the natural world	0	0	**1**	Conservationist
Biodiversity gain	Increase in species richness	**1**	0	0	Conservationist
Biosphere interaction	Interaction between people and nature Relation between locals and the Estate Different types of environments and ecology	0.2	0	**0.8**	Other
Carbon sequestration	Reduce inputs Carbon-based analysis Species diversity at the site Biodiversity presence	0.2	**0.8**	0	Other
Climate change resilience	Permanent habitat Connectivity to the landscape Locks in carbon	0.4	**0.6**	0	Conservationist
Climate mitigation	Reducing carbon inputs Carbon sequestration	0	**1**	0	Conservationist
Climate resilience	Protection of Downland ecosystem Fit for purpose in climate changed landscape	0.3	**0.7**	0	Conservationist
Community engagement	Local jobs and food People engaged in management	0	0.2	**0.8**	Producer
Democratic access	Unrestricted access for all	0	0	**1**	Conservationist
Diversity	Complexity, resilience and productivity As many native species as possible	0.4	**0.6**	0	Producer
Earth care	Reciprocal relationship between people and land Soil health and biodiversity No pesticides, herbicides, insecticides Biodiversity	0.2	0.3	**0.5**	Producer
Economic benefit	Cost–benefit analysis	0	**1**	0	Conservationist
Economic viability	Economically viable for landowner	0	**1**	0	Other
Ecosystem services	Focus on the whole suite of ecosystem services Services that are and could be provided	0	**1**	0	Conservationist
Education	The way we interact with the land	0	0	**1**	Other
Environmental concerns	Agrochemical and water use Specific management practices on the land	0	**1**	0	Producer
Fair share	Growing food, recycling nutrients Fair share with land ownership	0	**0.6**	0.4	Producer
Food production	Amount of food produced	0	**1**	0	Producer
Groundwater protection	Protecting city’s water supplies and purity	0	**1**	0	Other
Groundwater quality	Reducing risk to groundwater	0	**1**	0	Other
Habitat change	Preserve existing habitats Create new space for wildlife	**1**	0	0	Producer
Historical baseline	Historical period against which restoration is measured Most original habitat as possible	**0.9**	0	0.1	Producer
Justice	Fairness of proposed change	0	0	**1**	Other
Landscape	Quality of the view people will see	0	0.2	**0.8**	Producer
Locally sourced food	Supply of food to the city	0.1	**0.6**	0.3	Producer
Natural usefulness	Most naturally available uses of the land What does the land lend itself to doing most easily?	0.4	**0.5**	0.1	Producer
Nature recovery network	Quality and condition of individual pieces Connectivity Biodiversity and habitat resilience—climate crisis	**0.7**	0.3	0	Conservationist
Nature recovery network (2)	Healthy trophic pyramid Mosaic of habitats Diverse interactions from people Connectivity	**0.6**	0.1	0.3	Conservationist
Nature recovery potential	Restoring natural processes	**1**	0	0	Conservationist
People care	Giving people access to the land Public health Price to get onto land Exercise	0	0.3	**0.7**	Producer
Percent habitat created	Proportion of good quality habitat created	**1**	0	0	Conservationist
Recreation	Free to use Accessible	0	0	**1**	Other
Rental income	Ability to generate income for the landowner	0	**1**	0	Producer
Resilience	Extent land use can respond to climatic, biotic and other stresses	0	**1**	0	Other
Social benefits	Mental and physical health benefits from land use	0	0.2	**0.8**	Producer
Soil health	Structure, biodiversity, carbon Overall soil health Not purely agricultural perspective	**0.9**	0.1	0	Conservationist
Sustainable economic	Economic sustainability of land use over time	0	**1**	0	Producer
Sustainable environmental	Habitat Biodiversity Nature recovery Climate change	**0.8**	0.2	0	Producer
Sustainable social	Accessibility Education and information Full public access important, but impossible if want to enhance biodiversity	0	0.1	**0.9**	Producer
Sustainable land use	Future proofing of the site Relevant in the context of climate change Bringing agriculture, economy, forestry, community and environment together	0	**0.8**	0.2	Conservationist
Water protection	Reduction in water pollution	0	**1**	0	Conservationist
Wider public benefits	Public access and community use Local food growing	0	0.2	**0.8**	Conservationist

Keywords and phrases taken from the key features and description used in scoring the criteria and NFF scores (scores are based on key features and a broader description was provided by the participant). Scores in bold are the highest scoring NFF component; this is the group in which the criteria was placed for the ranking stage of the analysis

^a^Dr. Dan Danahar

To further explore the NFF as a tool to map social values of landscape change, the same exercise was carried out with an additional 67 criteria from a comparable study of Balfour et al. (2021) and Durrant and Ely (2022) (for mapping see supplementary information, S2). Balfour et al. (2021) carried out an MCM appraisal to assess how different sustainable food production and biodiversity conservation scenarios deliver the diverse needs of people and nature in South East England. For these additional criteria, the researchers were not present for the interviews and did not have access to the full transcripts. However, we were provided with an anonymized dataset containing the title, key features, and description for all criteria.

## Results

### Ranks

Three participants from the Downland Estate interviews defined their own land use options to be included in the analysis. One participant defined an option “Rewilding” and two defined “Community Farm”. Generally, these individual options were scored highly. Participants defining their own option did so to fit their personal vision of how the area should be used. For example, the “Rewilding” option was envisioned as “somewhere in between regenerative agriculture and passive rewilding” and was perceived as a more suitable strategy in the context of the Downland Estate than either of these core options.

**Fig. 4:**
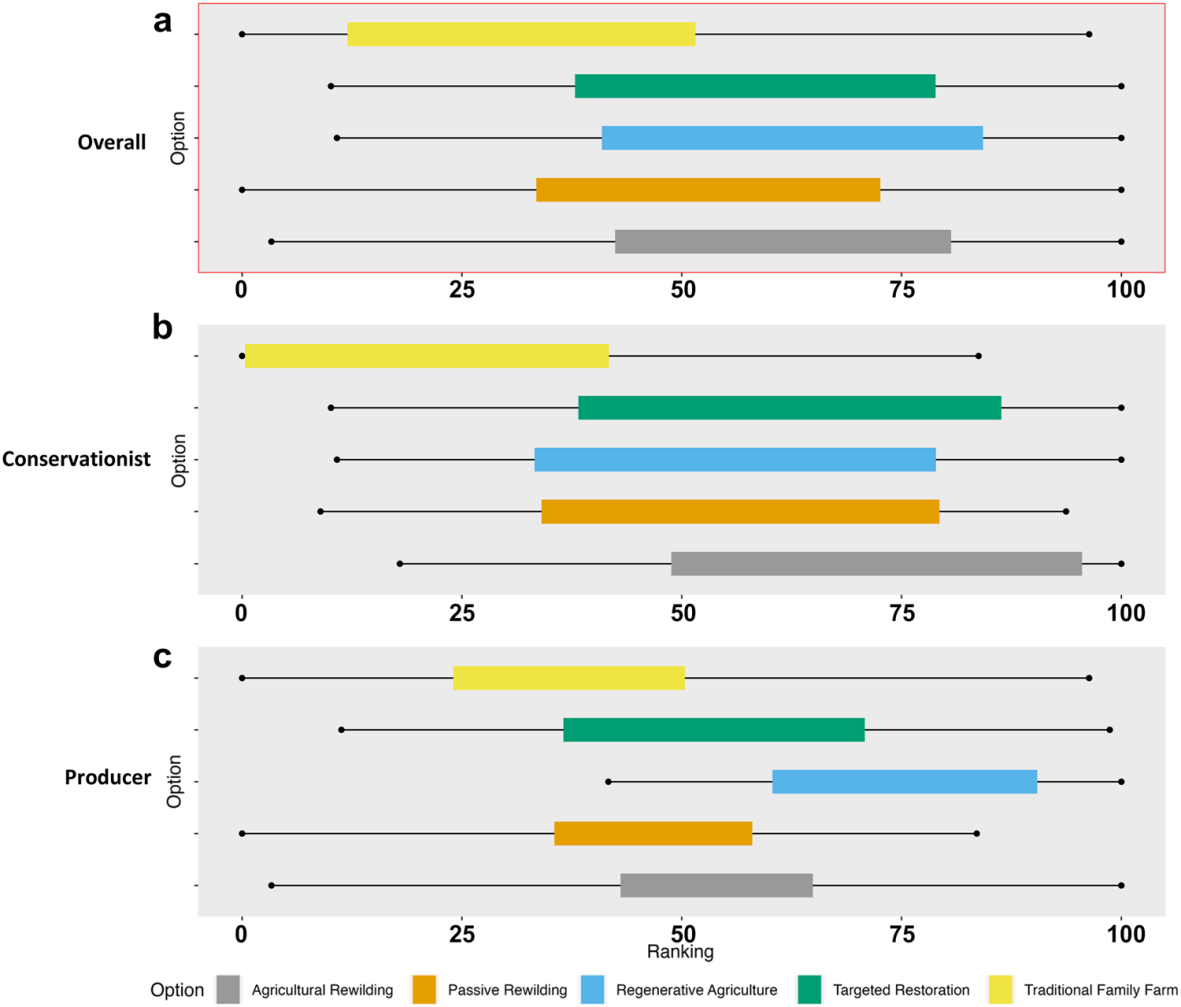
Overall aggregate rank chart for the core options from the MCM exercise. Ranks are calculated using pessimistic (hypothetical worst-case scenario) and optimistic (hypothetical best-case scenario) scores assigned by the 13 participants, using all criteria. The ‘rank extrema’ (black points) represent the full variability in the ranks assigned by different participants; the lowest point represents the most pessimistically scored individual criterion, while the upper point is the most optimistically scored criterion. The ‘rank means’ (colored bars) show the distribution of participants' ranks within this range—the lower value of the bar indicates the mean of the pessimistic ranks, and the upper end the means of the optimistic ranks (Coburn et al. 2019)

Across all value perspectives and core options, there was no clear nature recovery option that ranked the best: agricultural rewilding, passive rewilding, regenerative agriculture, and targeted restoration all performed strongly (Fig. 4). The exception was the status quo option, traditional family farm. However, this was less distinct for producers, as the high-ranking extrema bar shows that for certain criterion(s), this option is viewed to have the potential to perform well when scored optimistically. Looking between stakeholder groups, we can see that the overall weaker performance of traditional family farm was driven by conservationists scoring this option particularly poorly, while producers were also less optimistic about its performance against most, but not all, criteria.

### Nature for nature

**Fig. 5:**
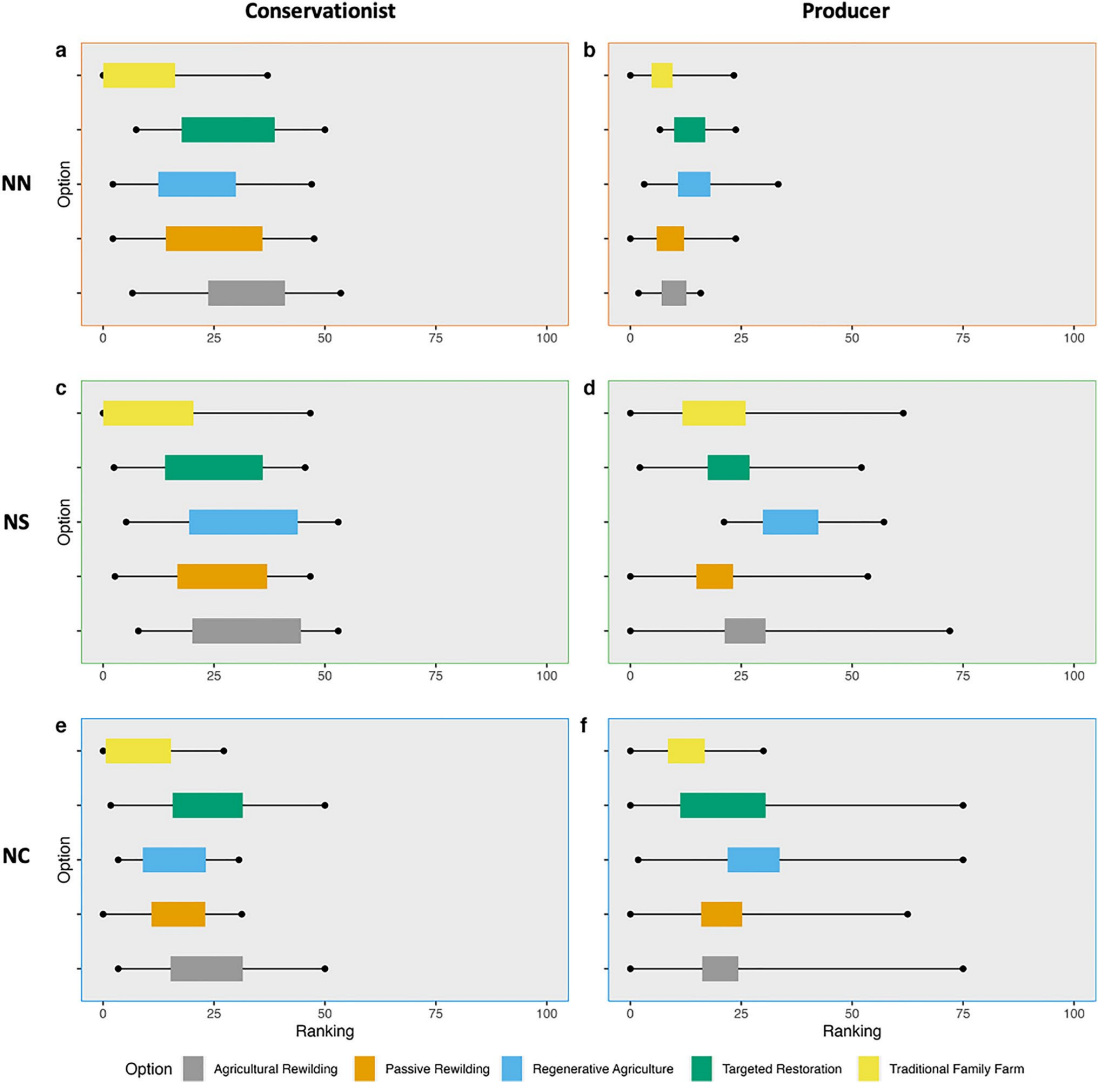
Aggregate rank chart for the core options from the MCM exercise, by stakeholder group: conservationists (5), producers (5). Ranks are calculated using only criteria from specific NFF perspectives. Rankings: **a** conservationists, NN criteria; **b** producers, NN criteria; **c** conservationists, NS criteria; **d** producers, NS criteria; **e** conservationists, NC criteria; **f** producers, NC criteria

Conservationists were most optimistic about agricultural rewilding for promoting biodiversity conservation under the NN value perspective (Fig. 5). The introduction of large-bodied species within the agricultural rewilding option was generally seen as preferable versus the passive rewilding option, since it would “make the ecosystem more diverse”. For producers, regenerative agriculture was the best-performing option across multiple criteria; however, the low pessimistic extrema suggests that for certain criteria it is perceived as performing poorly. Targeted restoration was also viewed to have high potential for a number of criteria when considering it optimistically. Across both stakeholder groups, traditional family farm was the worst-performing option; however, this pattern was less distinct for producers, where agricultural and passive rewilding were both generally scored poorly across NN criteria.

### Nature for society

For the NS perspective, regenerative agriculture ranked highly for both stakeholder groups (Fig. 5). It was seen as the best option to “produce a lot of food” and provide ecosystem services, while also being “very climate resilient”. For conservationists, multiple options were viewed optimistically in comparison to the traditional family farm option, which was perceived as the clear worst-performing option for NS criteria. It consistently scored low on climate-based criteria, and the negative implications of agrochemical usage was a key theme. For producers, there is more uncertainty in the rankings, reflected by wider extrema bars. Across a range of criteria regenerative agriculture is the highest ranking. Yet, alternative options also perform well for specific criteria, for example agricultural rewilding records the highest optimistic extrema score.

### Nature as culture, one with nature

For conservationists, agricultural rewilding and targeted restoration were, on average, the best-performing options for NC and traditional family farm was the clear worst-performing option (Fig. 5). This land use was perceived as often inaccessible, and not engaging the local community, “just a farmer and family”. Regenerative agriculture had the highest rank mean values for producers; however, the high-ranking extrema bars for multiple options suggest that, depending on the criteria assessed, they may also perform strongly. Traditional family farm and passive and agricultural rewilding were all generally scored low across NC criteria.

### Tenant farmers

When we consider just the responses of the two tenant farmers, traditional family farm was perceived to perform much better across criteria, and regenerative agriculture also ranked highly. The other land management options focused on nature recovery (passive rewilding, agricultural rewilding, targeted restoration) scored lower (see supplementary information figure, S2). There was the belief that the general public “find the current downs landscape to be attractive”, while rewilding would lead to an undesirable landscape. Their perception was that passive rewilding would tend towards scrubland. An example was cited of a local area left for 50 years that was a “messy scrub—worst-case scenario”. There were also economic concerns about the rewilding options: “if we rewild large areas of the downs, then the value of the land will decrease”. This was reflected by one participant assigning negative scores for passive rewilding, agricultural rewilding and targeted restoration under the “rental income” criterion. Optimistic scores for passive rewilding were given with the caveat that it would only succeed in scenarios where it was supported by subsidies, and for agricultural rewilding if meat was sold at a “super premium price”. By contrast, traditional family farm and regenerative agriculture were seen as both economically and environmentally sustainable land uses. Agricultural landscapes that included a “variety of habitat” were perceived as being the best for the environment. These options also scored highly for social criteria, with one participant highlighting the success of Open Farm Sunday (an annual farm open day, educating visitors about farming and the countryside) in engaging the local community.

### NFF Mapping

**Fig. 6:**
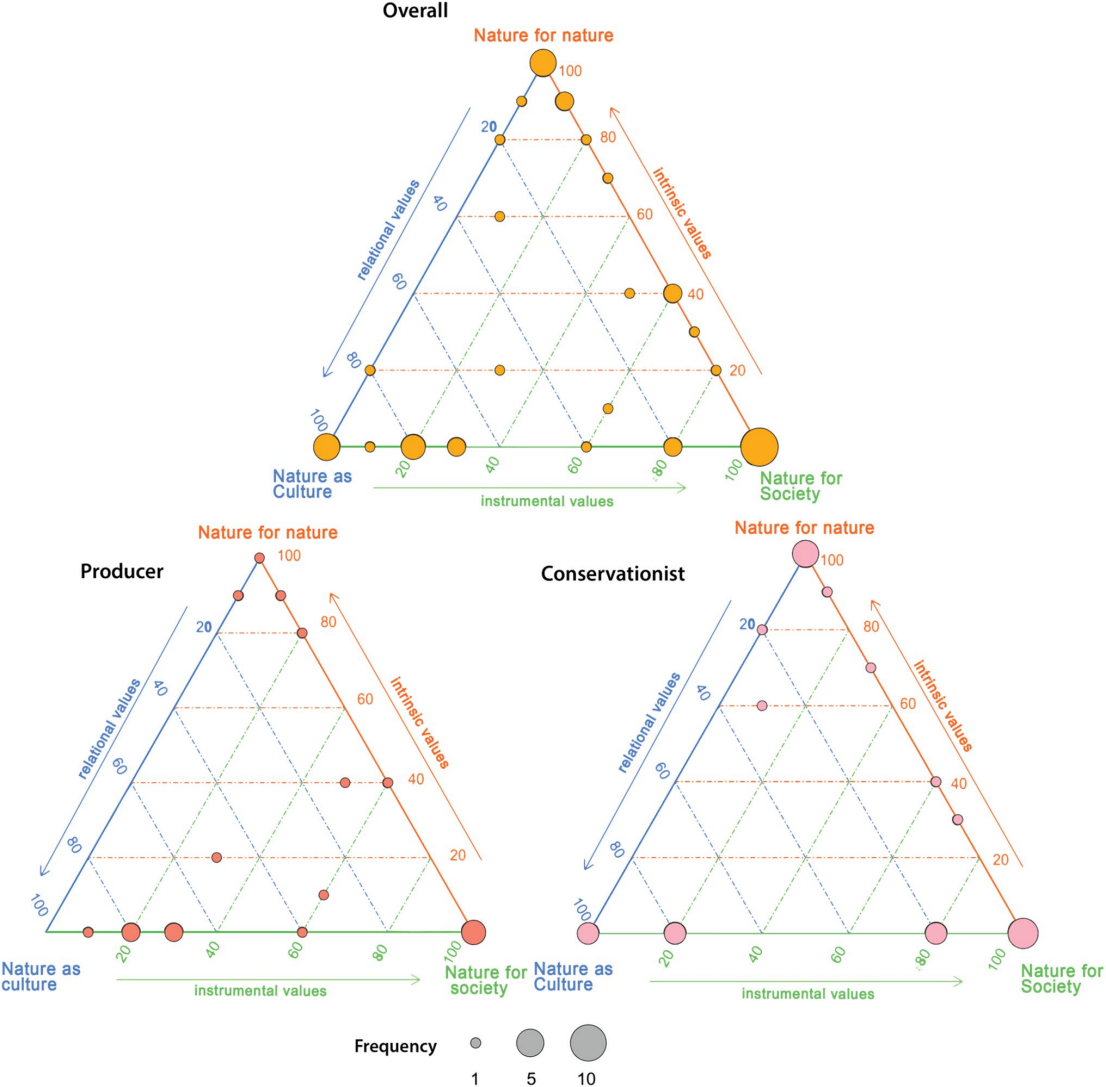
Mapping the participant-defined assessment criteria from MCM interviews onto the Nature Futures Triangle. Blue axis: NC; orange axis: NN; green axis: NS. Points represent mapped position of criteria. Size of the point equals the number of criteria mapped at that location. Top triangle, overall, maps all 47 criteria elicited from the Downland Estate MCM. Bottom left, producer, maps all criteria (18) defined by participants from the producer perspective; bottom right, conservationist, maps all criteria (19) defined by participants from the conservationist perspective

From our study, the 13 MCM interviewees defined a total of 47 criteria, of which 22 were directly mapped to a single NFF perspective (were scored 1, 0, 0 for either NN, NS or NC; Fig. 6). The most common criteria classification was NS, with 12 criteria situated purely within this perspective. 5 criteria scored solely for NN, and 5 for NC. Of the remaining criteria, the majority are located around the outer axes of the NFF triangle, meaning they contained elements of only two of the NFF perspectives. Few criteria were elicited that combined elements of NN and NC. A small number of criteria (4) are situated within the triangle interior (meaning they contained elements of all three perspectives).

## Discussion

### Nature recovery on the Downland Estate

The results of this study showed a diverse range of preferences for land use on the Downland Estate. Generally, participants seemed open to change and nature recovery, illustrating the success of the MCM approach in catalysing discussion in the context of landscape change. While producers generally favoured the regenerative agriculture option and conservationists ranked agricultural rewilding the highest, when we focus on specific NFF value perspectives, different options come to the fore.

The targeted restoration option performs strongly for NC for conservationists, yet it is not the clear best-performing option as agricultural rewilding also ranks highly, and for producers it ranks second to regenerative agriculture. This is surprising given local campaigns to restore chalk grassland (e.g. Brighton Downs Alliance 2022) and the fact it is the option most closely aligned with a “cultural landscape”. The similar high ranking of agricultural rewilding (for conservationists) and poor performance relative to regenerative agriculture (for producers) would suggest some element of human-involved food production within the landscape is viewed as culturally important. This is reflected in the nearby Knepp Estate, which was highlighted as an example when scoring agricultural rewilding by some participants in the study. While Knepp aims to restore natural ecosystem dynamics, there still exists elements of human control with respect to the animals on the site; however, this is focused towards conservation and animal welfare as opposed to traditional farming outputs (Dempsey 2021).

Despite the strong performance of alternative production options (regenerative agriculture and agricultural rewilding), the status quo traditional family farm option consistently ranked low, both overall and when focussing on specific NFF perspectives. Nevertheless, when tenant farmers were interviewed (who currently operate agricultural systems near or on the estate), there was a strong preference for this option. Chapman et al. (2019) suggest that strong attachment to a status quo (here traditional farming on the site, where “people connect with the countryside, and “the public prefer the open landscape”), or community norm (Nassauer et al. 2009), can hinder ecological restoration. We observed clear misalignment in value systems between tenant farmers and the more hands-off nature recovery options (passive and agricultural rewilding). The respective high ranking of regenerative agriculture by the tenant farmers suggests they would be more open to making smaller changes in land management, rather than those perceived as more drastic alterations from the status quo.

The strong performance of both production options (traditional family farm and regenerative agriculture) among tenant farmers may be driven by relational values (Drenthen 2009; Chapman et al. 2019), while analysis of the qualitative data also shows that attachment to production options is grounded in the concept of food security: “Food security, self-sufficiency as a nation is important… As a nation can we afford to take massive areas out of production and importing from abroad?” Among the community producers interviewed, food production and food security were also key issues reflected in criteria definitions. However, some saw this as a problem isolated from management of the Downland Estate: “there is absolutely a concern here about where food should come from, how will a farmer make a living if we are going to import everything? But food production shouldn’t be a conversation on the Downland Estate, but maybe just about everywhere else, where land is fertile and productive”. Instead, they focused more on the idea of active community involvement in the production of “high-quality produce for the local market”. While the status quo management option was seen as damaging to the environment and climate—an opinion coherent with the broader literature (Landis et al. 2008; Carlsson-Kanyama and González 2009)—regenerative agriculture was perceived by producers as a sustainable and innovative method to produce food for local markets: “I would like to see a lot of small farms re-established over the next 50 years, but in an agri-ecological way only. We don’t have time to not farm in this way”. This point was further illustrated by the concluding remark of one participant that “the city of the future feeds itself”. To what extent regenerative agriculture actually offers such a solution is debatable (Giller et al. 2021), but it was clear this option was viewed favourably by participants.

While there is considerable overlap between groups in the general position of criteria on the NFF triangle (Fig. 6), we observed a split between criteria focus. For conservationists, there were considerably fewer criteria related to food production. Instead, they often defined criteria related to a wider suite of ecosystem services and climate change mitigation. This difference in opinions between stakeholder groups may in part be driven by pre-conceived notions of how the Estate should be managed. The rewilding options were perceived as not being suited to large scale production, and thus for producers that were interested in food security, these options scored poorly. Moreover, for some respondents, the issue of food production actually drove low scores for the rewilding options on criteria that were not directly linked to food production. For instance, one participant scored passive rewilding low for their “rental income” criterion as it was “the least productive of the options” and for their “habitat change” criterion as it “removes significant areas of arable habitat”.

The two rewilding options varied considerably in their performance across stakeholder groups and NFF value perspectives. Between participants, it was clear “rewilding” was a polarizing term, eliciting varying responses. For example, under the passive rewilding option, the tenant farmers interviewed questioned “where the money would come from” and predicted a “messy, scrub landscape that would be the worst-case scenario”, and a “landscape that people would not appreciate as they do the Downlands at the moment”. Value for tidy, managed landscapes, is one that has been widely found among farmers (Dessein and Nevens 2007; Schneider et al. 2010). The change from well-managed agricultural land, to land abandonment in the passive scenario, could be perceived as a transition from NC to NN values. Agricultural rewilding was perceived as an option closest to “winding back the clock”, which “can be attractive to environmentalists”, but is contrary to what the general public desire—“open landscape”. By contrast, conservationists generally saw the rewilding options as a means to “encourage nature-led recovery”. While there were concerns around the unpredictability of passive rewilding, the inclusion of herbivores in the agricultural rewilding option was seen as key to “creating more niches”, and “making a mosaic of habitats within the ecosystem”—this preference could be driven by the well-publicized successes of the nearby Knepp Estate (Dempsey 2021).

The MCM methodology, in combination with the NFF, can help highlight pathways to achieving maximum gains for society, nature, and culture, which may be ignored in more conventional appraisals. The relatively strong ranking of a number of the nature recovery options defined suggests that a diverse mix of these land uses could be the best way forward to deliver against the range of criteria selected for the Downland Estate as a whole. This is also reflected in the qualitative data. For example, one participant suggested their additionally defined “Rewilding” option would best “work alongside regenerative agriculture”. Such multi-functional use of ecosystems can be ecologically, socio-culturally, and economically advantageous (de Groot et al. 2010).

However, a number of high performing options for certain perspectives also elicit strong negative feelings from other stakeholders and groups. For instance, agricultural rewilding performs well for the conservationist group under NN criteria, but performs relatively poorly for producers. This presents an interesting policy dilemma: is the aim to minimize the number of people who are unhappy with a decision, which is likely best achieved by pursuing a single use on the estate that scores consistently well across perspectives (e.g. regenerative agriculture)? Or, is the aim to meet all criteria and provide the favoured sites for all stakeholders by supporting a mosaic of uses, even if this means all stakeholders also have a site they are opposed to?

### Nature Futures Framework

Our novel application of the NFF, in combination with the participatory MCM approach, allowed us to explore the potential of the NFF as a tool to map stakeholder values (as invited by IPBES 2022). Solely applying scenario-based approaches may conceal novel narratives and contextual information (Sitas et al. 2019); thus, it is important that approaches are flexible and able to incorporate the diverse ways in which stakeholders value nature. In our mapping exercises for the criteria from the Downland Estate MCM, we were able to map all criteria to the NFF.

Balfour et al. (2021) and Durrant and Ely (2022) used the ecosystem services framework to map criteria elicited from a comparable MCM study, using the following groups: supporting and regulating ecosystem services (SRES), provisioning ecosystem services (PES), and cultural ecosystem services (CES). Whilst they found that the majority of their criteria could be categorized using the ecosystem service framework, such an approach was also limited, as it lacked sensitivity and was unable to account for site-specific considerations (Durrant and Ely 2022). Of the 67 criteria defined in their study, they were able to map 49 (70%) within their defined ecosystem service categories. The unclassified criteria largely fell within two key themes: desirability and viability. While these criteria were often quite personal to the given participant, and thus more difficult to conceive from our secondary analysis of the criteria having not taken part in the interview process in which the criteria were elicited, we found it was still possible to map them to the NFF (see supplementary information, S2).

“Land Use Intensity” was one desirability criteria that Durrant and Ely (2022) were unable to map to the ecosystem service framework. This criterion focused on efficient use of the land in order to meet both agricultural (NS) and biodiversity (NN) outcomes, a concept that is difficult to consider within the bounds of traditional ecosystem service assessment. However, within the NFF framework, one can use the overlapping nature of the perspectives to classify this criterion. First, the idea of efficient land use and agricultural output is a societal problem, thus we score the criteria highly for NS; however the criterion also relates to biodiversity, and thus is also scored for NN—leading it to be mapped along the NS (0.6), NN (0.4) axis. Viability criteria focused on economic or financial issues (Durrant and Ely 2022) and were defined by a number of participants. Generally, such criteria could be classified under NS as they are ultimately concerned with human benefit from the land use, which is financial security over time. Using the NFF, we were able to categorize 100% of the criteria from both our study and that of Durrant and Ely (2022).

In addition to not being able to completely map criteria to the ecosystem service framework used, Durrant and Ely (2022) highlighted additional criteria that were not clearly defined within the conventional ecosystem service framework. They found that the SRES definition used was limiting when attempting to classify criteria related to biodiversity or soil (as articulated and valued by the participants). The stock-and-flow framing of people–nature relationships within the ecosystem service framework has been criticized as being inadequate for representing the broad range of human–nature values (Norgaard 2010)—most notably services that are not amenable to biophysical or monetary metrics (Chan et al. 2012). Within the NFF framework, the cross-cutting nature of participant-defined criteria are less problematic. NFF perspectives are inherently overlapping in space, which meant there was more flexibility in classifying criteria. For instance, biodiversity criteria are often mapped to both NN and NS perspectives, as they contain not only social values, but also the concepts around the benefits that this biodiversity will bring to people, e.g. pollination services. This overlapping nature of the NFF better incorporates the plurality of ways in which people assess the  nature recovery value than the ecosystem service approach, in which the more rigid nature of the categories (SRES, PES, CES) can make it difficult to classify criteria for which the participant description covers a broad range of values and issues.

To best develop land management practices within the urban and peri-urban context, we need to incorporate a wide suite of stakeholders to ensure equitable environmental outcomes. For instance, indigenous peoples may hold different normative positions in how land should be managed (Pascual et al. 2021). MCM in combination with NFF offers the potential to better elicit such values, in a comparable and quantifiable way, as it allows novel options for landscape management to be investigated across a diverse set of stakeholders (Elsawah et al. 2020; Quintero‐Uribe et al. 2022). While many criteria defined in our study could fit into conventional assessments, there were also a number of more novel criteria that contained elements of more than one NFF perspective (Table 2). The structure of the NFF triangle allows it to serve as a boundary object for opening and holding a plurality of value perspectives (Palacios-Abrantes et al. 2022) and meant it was still possible to categorize and classify these criteria, which are often very dynamic and non-linear. Our application of the NFF highlights the need to implement frameworks that are adaptable in order to reflect the complexity inherent within social–ecological systems.

Analysis of criteria, and their underlying patterns, can be especially useful for landscape planning and ensuring equitable social outcomes from nature recovery projects. This follows from the recommendations of Mansur et al. (2022) with regard to the need to consider the diverse perspectives relating to a site that is of high value to local populations. Nevertheless, there could be limitations with the methodology used in this study, and we stress it should not be seen as a catch-all approach for assessing stakeholder values in landscape planning. The methodology may still underrepresent specific values; for instance, we found relatively few criteria that contained elements of both the NN and NC perspectives. This could be contextual, in that these values are less pronounced in the given peri-urban and/or agricultural contexts. Alternatively, these values could be more difficult to articulate in MCM, and thus not defined as criteria by participants, which would thus require a different approach for them to be brought to the fore. Future applications of the methodology used in this study, in alternative contexts, could help to better answer this question.

## Conclusion

The results of this study highlighted the diverse range of ways stakeholders respond to the prospect of nature recovery and landscape change. The detailed quantitative and qualitative data elicited in the MCM approach can help to assess co-benefits and potential areas of conflict between stakeholder groups. For example, we highlight potential conflicts between and within stakeholder groups regarding food production. While some participants see food as an integral element of future land use, others place greater important on recovering nature, tackling climate change, and community connection. Land use rankings vary across NFF perspectives and between stakeholder groups. This suggests a mosaic of land uses that incorporates all suggested management options, to create a diverse Downland Estate, would be a successful future management strategy to meet social, natural, and cultural needs. The combined approach of using MCM and NFF has successfully highlighted the likely importance of diverse land use and management to meet the varied needs of people and nature. The next challenge is addressing the geography of where and how much land is given to different management.

The NFF provided a broad and flexible framework for categorizing the social values elicited from both MCM studies, outperforming the conventional ecosystem service framework. The MCM interview process, in combination with the NFF framework, has significant scope for future applications, particularly in highlighting and elevating underrepresented stakeholder values. This should take place across different contexts, to further bring to the fore underrepresented stakeholder values, and to help elicit points of conflict between key stakeholder groups.

## Supplementary Information

Below is the link to the electronic supplementary material.

Supplementary file1 (DOCX 2002 KB)

## Data Availability

Not applicable.

## Footnotes

1 multicriteriamapping.com.
