# Supplementary material for: Diverse approaches to nature recovery are needed to meet the varied needs of people and nature
Source: Sustain Sci. 2023 May 25:1–17. Online ahead of print. doi: 10.1007/s11625-023-01337-w (PMC10209942; doi:10.1007/s11625-023-01337-w)
Supplement: Supplementary file 1 — Supplementary file1 (DOCX 2002 KB) [file 11625_2023_1337_MOESM1_ESM.docx]

Diverse approaches to nature recovery are needed to meet the varied needs of people and nature

**Supplementary information**

S1

Participant briefing document for the Downland Estate MCM

BRIEFING FOR PARTICIPANTS

Assessing nature recovery options on Brighton’s Downland Estate

*How do we create a landscape that meets the diverse needs of people and nature in Brighton?*

**Introduction**

This research aims to assess the contrasting nature recovery options on the Brighton and Hove City Council’s rural Downland Estate that could help meet the needs of people and nature. We wish to gain a detailed understanding of how a diverse group of expert stakeholders assess alternative nature recovery options on the estate.

These results offer the opportunity to help guide future management and answer the following questions: is a single nature recovery option best suited for delivering the needs of people and nature in the region, or are a diverse set of approaches needed? Do experts with a variety of perspectives assess nature recovery options similarly? What are the costs and benefits of different nature recovery? We hope this research may contribute useful insights to those considering the costs and benefits of different land uses across the estate as a whole, as well as to individual land-owners and managers considering alternative land uses.

The Downland Estate consists of approximately 5,200 hectares of land, with 94% of it within the South Downs National Park. The entire site is characterised by chalky, silty loam soil type of intermediate depth. Current land use across the site is 78% farmland, with 9% woodland and land for recreational activity is also important. There are 40 wildlife sites, 5 local nature reserves, 2 Sites of Special Scientific Interest, 1 special area of conservation, and 1 national nature reserve. Primarily, the land consists of grade 3 (good to moderate) and grade 4 (poor quality) agricultural land. Development of a new whole estate management plan is currently underway and will support future decision making across the breadth of the site.

Specifically, for this research, we are asking you to assess a variety of nature-recovery options that could be implemented on a hypothetical 200ha (3.8%) contiguous land holding on the estate which is currently being used for arable production.

By agreeing to participate in an MCM interview, you are agreeing to assess a suite of options that represent contrasting nature recovery options for the Downland Estate. Your appraisal will be considered in conjunction with a number of other appraisals carried out by experts with different perspectives on the issue at hand. The next steps in the process are explained at the end of this briefing.

Map of Downland Estate boundary


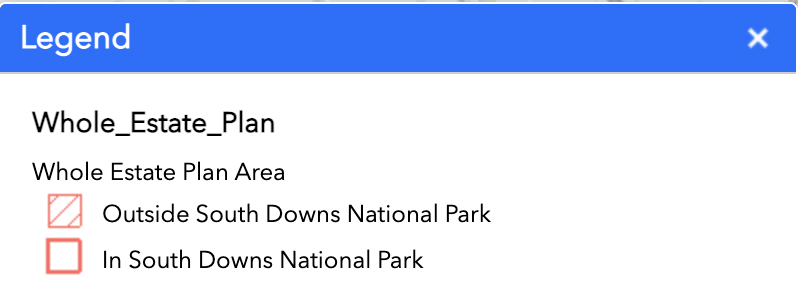


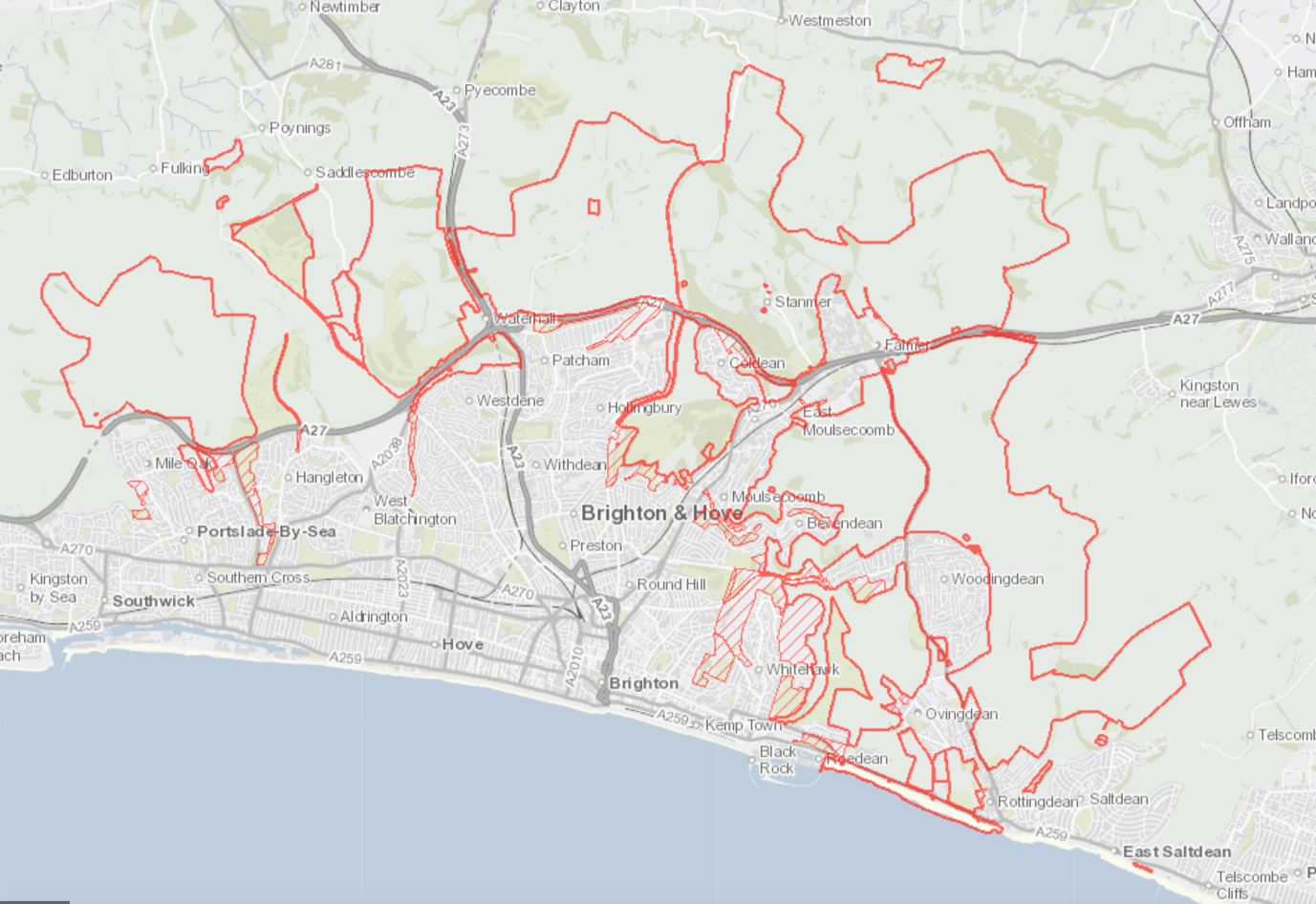


<https://bhcc.maps.arcgis.com/apps/webappviewer/index.html?id=e3c872deb3c9464fb97b3ee10731b944>

**Nature recovery options**

The options described below (Table 1) represent contrasting options for nature recovery on the Downland Estate. They take the form of different options for managing a hypothetical portion of the Downland Estate that has the following characteristics: **200 ha in size**, **chalky soil type**, **currently used for arable agricultural production**, **within walking distance of the city outskirts**.

We are exploring a hypothetical scenario where a site owner and manager is open to considering a number of innovative nature recovery options and associated business opportunities and human uses that are consistent with Brighton and Hove City Council’s vision for the estate. The key management actions for each option are summarised in Table 1. Of particular interest is how expert stakeholders perceive the opportunities and risks associated with each of these nature recovery options.

A glossary of key management terms is provided at the end of this document.

Table 1. Summary of land management options

| Land management option | Description |
| --- | --- |
| **Traditional Family Farm**  (This option best replicates current land use on the site) | This option is focussed on agricultural production. The site will be a mix of arable fields (primarily producing spring barley and winter wheat), with agrochemicals used to increase yield. Ideas of nature recovery are not at the forefront of land management plans.  All farming at the site complies with existing environmental standards (under Red Tractor certification). |
| **Regenerative Agriculture** | Regenerative agriculture is an alternative means of food production. It is based on the following key processes: no, or low, external inputs, and increasing the efficiency of on farm inputs; integration of livestock in the agricultural system; no or minimal use of synthetic fertilisers and pesticides; and reduction in, or elimination, of tillage.  At this site all livestock production will be certified to the Soil Association organic standard.  Alongside meat production, organic fruit and vegetable production, such as the planting of apple orchards, will be prioritised. |
| **Agricultural Rewilding** | This option comprises a significant shift towards a landscape governed by natural processes. Arable production at the site would cease, and free roaming heritage cattle and fallow deer are introduced, with the site boundary fenced (that allows public access through pre-established public rights of way). Seasonal introductions of heritage pigs.  Over time human intervention would be minimal, with emphasis on allowing plant communities to develop naturally. Rather than working to prevent disturbance regimes, such as wind damage, these are accepted as natural processes that shape the landscape.  The opportunity for food production in this scenario is associated with the management of the introduced large mammal populations. |
| **Targeted Restoration** | This option is targeting the restoration of chalk grassland as a locally important habitat. Arable production at the site would cease. After the harvesting of the last crop a sterile seedbank is created by using an herbicide to remove arable weeds. The land is then cultivated, and sowed with locally sourced, native seeds.  Hay cropping in the second and third year of the restoration program would be used to facilitate nutrient reduction in the soil and the flowering and seeding of chalk grassland flora. Managed grazing regimes would be implemented, primarily with sheep grazing in the autumn and winter. Cattle may also be used to achieve restoration goals if required. |
| **Passive Rewilding** | This option comprises a significant shift towards a landscape governed by natural processes, this means minimal human intervention and emphasis on allowing plant communities to develop naturally. There are no species reintroductions. Instead, a passive approach is taken to nature restoration.  Rather than working to prevent disturbance regimes, such as wind damage, these are accepted as natural processes that shape the landscape.  There is no agricultural production undertaken in this option, however non-extractive businesses are possible, and recreation is welcomed. |

**Next steps**

Once you have familiarised yourself with the five options, the next step is to **think about the main factors you may use to appraise these nature recovery options** as potential land management strategies for the Downland Estate. You may also define your own option, that can be included within the analysis, if you believe that a critical strategy has been missed.

The factors you select for appraisal will then become your criteria for assessing the five options against. In the interview you will be asked to define these criteria in a comparable manner, providing a title and key feature for each. For instance, if the overarching goal was to choose a design for a new building in the town centre, then criteria against which to appraise different options for achieving that goal might include ‘time to build’, ‘cost’, ‘aesthetic value’, and so on. **There is no set number of criteria expected, however 3-7 criteria tend to be a manageable number**. Any more may make the process too long.

Once you have defined your criteria, **the next part of the interview will involve scoring each nature recovery option for each criterion**. Rather than giving discrete scores (e.g., 50 out of 100), you will be asked to provide scoring ranges (e.g., 40-60 out of 100) and to discuss the optimistic and pessimistic assumptions (or different conditions) under which you would expect the performance to vary. If this sounds confusing or complicated now, don’t worry too much as it will make more sense in practice. This information is for the benefit of those of you who like to know what they’ll be asked to do in advance, but it is not necessary for you to completely understand it now as there will be time to talk it through in the interview.

**The final stages of the interview will be weighting your criteria, to provide an indication of their relative importance or relevance in your view, and reviewing your final ranking of the options once the appraisal is complete**. The overall aim of the MCM exercise is to “explore the ways in which different pictures of strategic choices change, depending on the view that is taken – not to prescribe a particular ‘best choice’” (MCM Manual V2 page 9). It is the reasoning behind the scores and ranks that matter – and how they compare across different participant’s appraisals – more than the scores themselves.

For further information please contact ____________:

_________@__________

**Glossary**

| External inputs | Artificial chemical inputs and mechanisation based on fossil fuels |
| --- | --- |
| Livestock integration | Mixing production of crops and livestock |
| Red Tractor | Farming certification scheme, covering everything from livestock production, to fresh produce and crops |
| Tillage | Preparation of land to grow crops |

S2


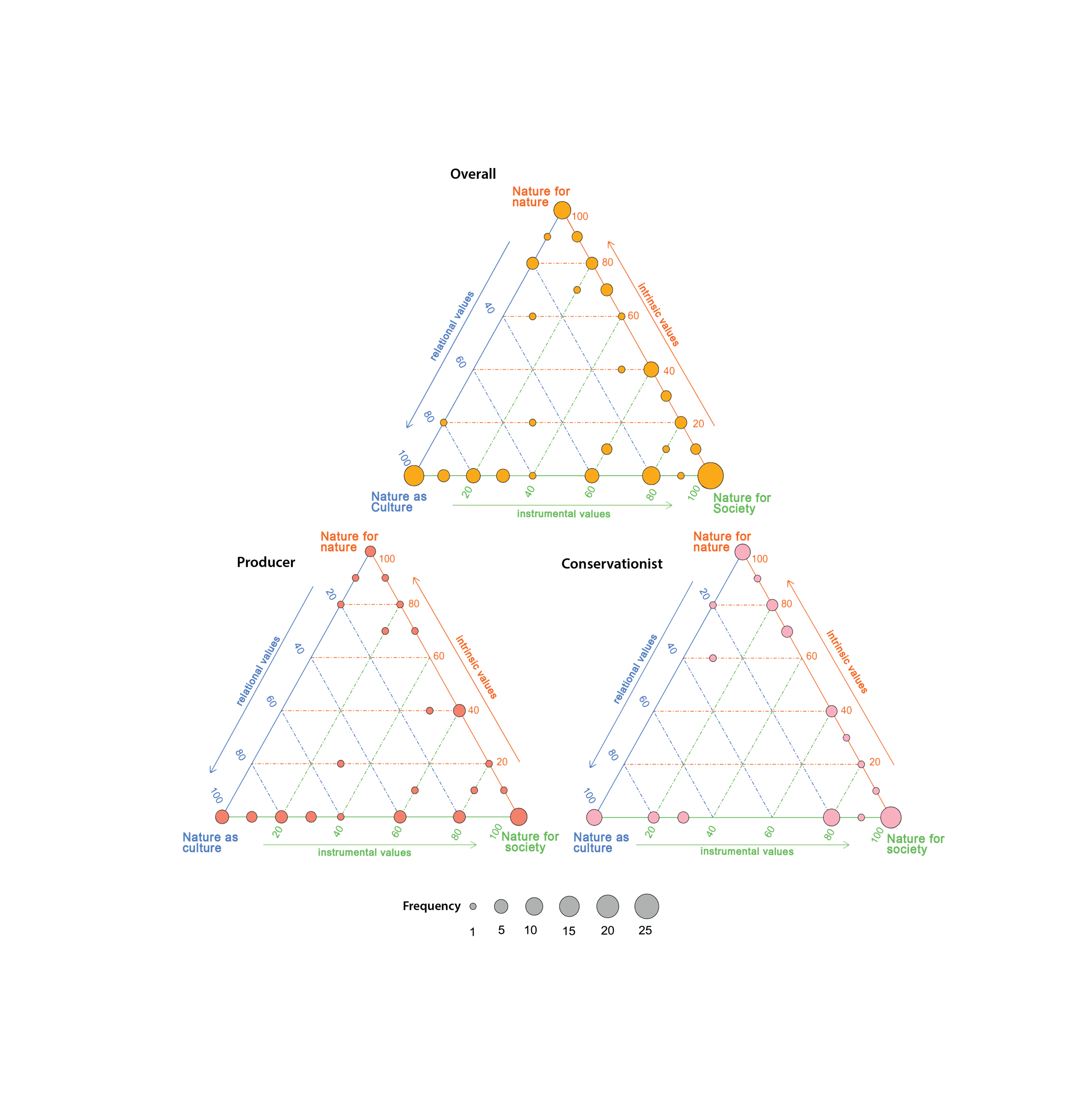


S3

Scenario ranking by tenant farmers


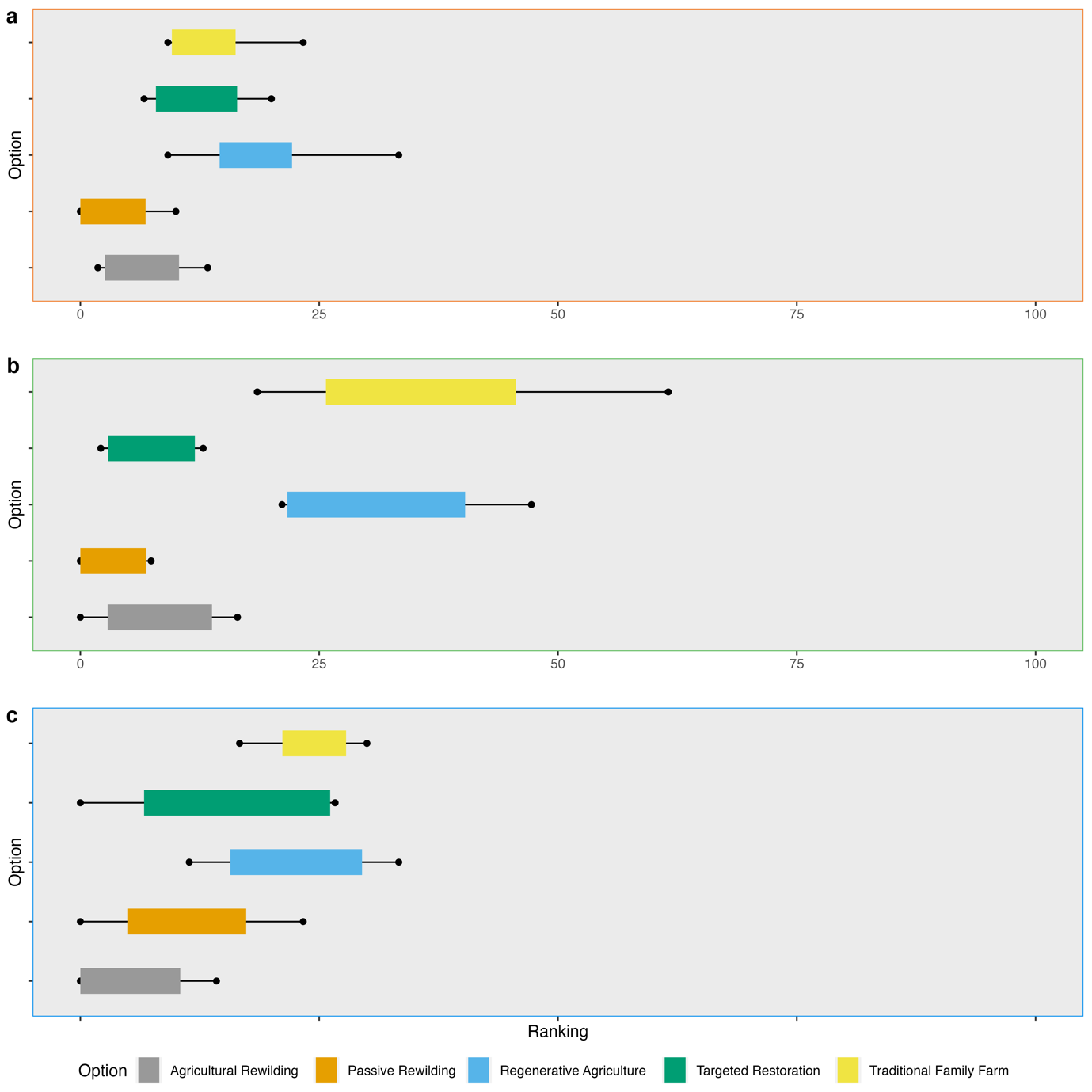


*Aggregate rank chart for the core options from the MCM exercise, solely for the 2 participants defined as tenant farmers within the producer group. Ranks are calculated only using criteria from specific NFF perspectives. Rankings: a) NN criteria; b) NS criteria; c) NC criteria*
